# An Analysis of US Academic Medical Center Websites: Usability Study

**DOI:** 10.2196/27750

**Published:** 2021-12-21

**Authors:** Jonathan James Gale, Kameron Collin Black, Joshua David Calvano, Edwin Lauritz Fundingsland Jr, Deborah Lai, Sara Silacci, Shuhan He

**Affiliations:** 1 Rocky Vista University College of Osteopathic Medicine Parker, CO United States; 2 Division of Psychology and Language Sciences University College London London United Kingdom; 3 Center for Innovation in Digital HealthCare Massachusetts General Hospital Boston, MA United States

**Keywords:** website usability, digital health, health care website, academic medical center, usability testing, web crawler

## Abstract

**Background:**

Health care organizations are tasked with providing web-based health resources and information. Usability refers to the ease of user experience on a website. In this study, we conducted a usability analysis of academic medical centers in the United States, which, to the best of our knowledge, has not been previously carried out.

**Objective:**

The primary aims of the study were to the following: (1) adapt a preexisting usability scoring methodology to academic medical centers; (2) apply and test this methodology on a sample set of academic medical center websites; and (3) make recommendations from these results on potential areas of improvements for our sample of academic medical center websites.

**Methods:**

All website usability testing took place from June 1, 2020, to December 15, 2020. We replicated a methodology developed in previous literature and applied it to academic medical centers. Our sample included 73 US academic medical centers. Usability was split into four broad categories: accessibility (the ability of those with low levels of computer literacy to access and navigate the hospital’s website); marketing (the ability of websites to be found through search engines and the relevance of descriptions to the links provided); content quality (grammar, frequency of information updates, material relevancy, and readability); and technology (download speed, quality of the programming code, and website infrastructure). Using these tools, we scored each website in each category. The composite of key factors in each category contributed to an overall “general usability” score for each website. An overall score was then calculated by applying a weighted percentage across all factors and was used for the final “overall usability” ranking.

**Results:**

The category with the highest average score was technology, with a 0.82 (SD 0.068, SE 0.008). The lowest-performing category was content quality, with an average of 0.22 (SD 0.069, SE 0.008). As these numbers reflect weighted percentages as an integer, the higher the score, the greater the overall usability in that category.

**Conclusions:**

Our data suggest that technology, on average, was the highest-scored variable among academic medical center websites. Because website functionality is essential to a user’s experience, it is justified that academic medical centers invest in optimal website performance. The overall lowest-scored variable was content quality. A potential reason for this may be that academic medical center websites are usually larger in size, making it difficult to monitor the increased quantity of content. An easy way to improve this variable is to conduct more frequent website audits to assess readability, grammar, and relevance. Marketing is another area in which these organizations have potential for improvement. Our recommendation is that organizations utilize search engine optimization techniques to improve their online visibility and discoverability.

## Introduction

### Background

A medical center’s website is often the first point of contact with the public; its initial impact is responsible for returning users and attracting new visitors [1,2]. It has the potential to be the first step in improving patient satisfaction as well as attracting new patients [3]. In a time when information is expected to be readily available, medical centers use their websites as a key tool for both patient communication and education [4-6]. The users expect to find current and reliable information on websites that are easily accessible in order to make health-related decisions [7]. With many sources available (eg, WebMD), medical centers are looking to improve their internet presence to better engage with potential consumers [3].

### Website Usability

Improving website usability is a noteworthy way in which medical centers can improve their internet presence to attract and retain more users and thus reach a larger audience with accurate and reliable information. Usability goes beyond surface-level design [8] but refers broadly to a product’s “user experience,” such as ease of navigation or encountered problems within a website [9]. It addresses the question of how easy or pleasing a website is to use (which can influence how many users engage with it), the level of engagement, and a website’s ability to achieve other objectives. When users are not able to easily access and use a website, they are unlikely to continue using that given source. Alternatively, improved usability can enhance the reach of a website. For this reason, websites are facing the increasing need to conform to user expectations, desires, and requirements [10,11]. Various industries have established standardized guidelines for accessibility, content, marketing, and technology in order to improve usability [12-14].

### Usability for Academic Medical Centers

Studies have sought to apply usability analyses to e-commerce, e-government, mobile news apps, and library websites [15-18]. In health care, other studies have looked at usability for hospitals, children’s hospitals, digital health centers, residencies, and cancer center websites [3,19-21]. However, to our knowledge, no studies of usability have been conducted exclusively on academic medical centers in the United States, which included all websites falling under the academic medical centers’ domain. Academic medical centers are the intersection of health professional schools, patient care, and academic research. Since an academic medical center comprises numerous institutions that function on their own part to be a part of a greater whole, they play a key role in the advancement of medical care [22]. Web presence is the way in which an academic medical center can demonstrate its advancements in their health professional schools, patient care, and academic research. It is important for these organizations to utilize usability metrics to not only improve user experience, but to represent themselves well as leaders in innovation.

### Objectives

The primary aims of the study were to adapt a usability scoring methodology to academic medical centers; to apply and test this methodology on a sample set of digital health center websites; and to make recommendations from these results on potential areas of improvements for our sample of academic medical center websites.

## Methods

### Sample Selection

**Figure 1:**
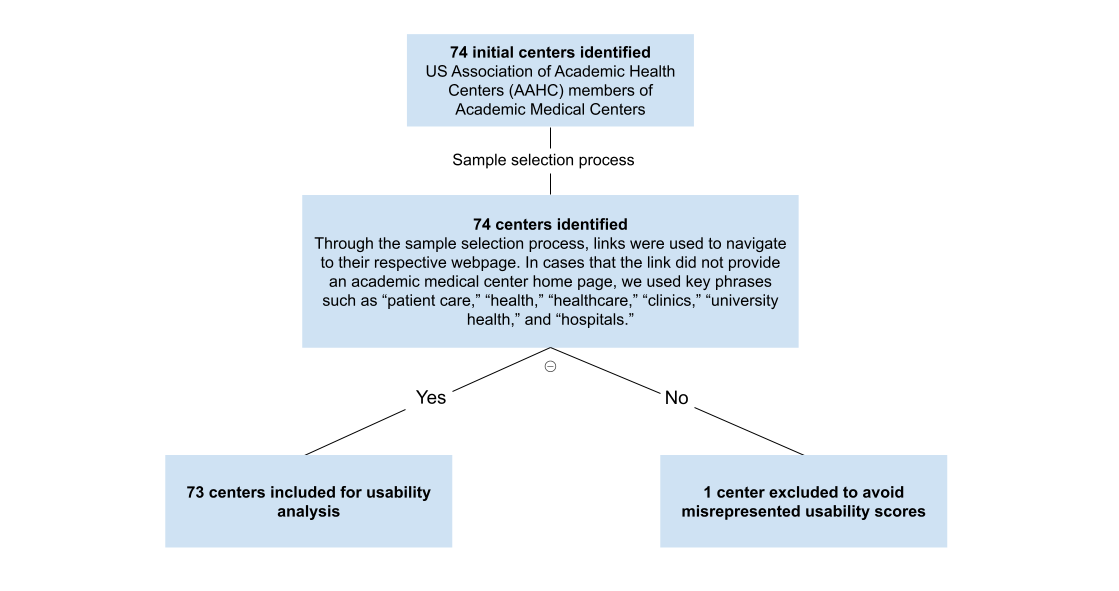
Sample selection criteria for academic medical center websites.

Our focus was on academic health centers in the United States. Indexing every academic medical center in the United States was not in the scope of our review; rather, this study focused on 74 academic medical centers listed on the members page of the Association of Academic Health Centers (AAHC) [23]. A link was provided for each of the academic medical centers listed, and the links were used to navigate to the appropriate academic medical center’s page. Some of the links navigated to the affiliated university’s website; therefore, terms such as “patient care,” “health,” “healthcare,” “clinics,” “university health,” and “hospitals,” were used to find the appropriate academic medical center’s main webpage. One link was removed from analysis, the University of California System, as this link provided a list of academic medical centers in California that were already included in the current analysis. This provided a total of 73 academic medical centers that were used for usability testing (Figure 1).

### Overview

All website usability testing took place from June 1, 2020, to December 15, 2020. We began by adapting a methodology developed in previous health care website ranking literature [3,19,24]. We kept the weighted percentages from the previous studies and applied specific formulas to those calculated percentages to create a relative scale for comparing usability scoring (Multimedia Appendix 1). Of note, the previous methodology used a consistent scoring system that used the relative maximum as the reference point of 1 so that every usability test fell between 0 and 1 [24]. However, we found that one of these tests did not follow this format, so our methodology improved the previous testing and applied the same reference range to the values.

Usability was sorted into the following four broad categories in order to ensure quantifiable, objective, and actionable recommendations for the websites: (1) accessibility: the ability of those with low levels of computer literacy to access and navigate the hospital’s website; (2) marketing: the ability of websites to be found through search engines and the relevance of descriptions to the links provided; (3) content quality: grammar, frequency of information updates, material relevancy, and readability; and (4) technology: download speed, quality of the programming code, and website infrastructure [3,19].

Each of these categories represents areas of usability in which academic medical centers can communicate more effectively with their audiences. The factors that contribute to each category were originally discussed by Huerta et al [3]. To add to the comprehensive nature of this study, we further defined these categories by utilizing support tools that bolster the credibility and reproducibility of the results of this analysis. These tools were chosen based on their ability to address each of the individual factors in the category criteria and their availability for public ease of use and implementation.

### Analysis

All of the websites were analyzed using a set of established usability tools. These tools were chosen based on their ability to specifically assess the individual factors selected. The tools were also chosen based on their ability to meet industry standards for evaluating the selected factor and for their relative ease of use. The process for utilizing each tool was based on the tool’s specific instruction manual. The authors collaboratively ensured the proper use of each tool and the proper value to be recorded. There were 2 researchers who collected data, and each underwent specific training on utilizing the suite of analytic tools and data entry. Questions and discrepancies were addressed by the project supervisor as they arose. Factors such as speed, which can vary from second to second, were averaged across 2 separate tools to provide the most accurate values possible. Overall, all the tools were run on a total of 2 computers to minimize as many outlying technology errors as possible. The selected tools can be viewed in Multimedia Appendix 1.

We began building a database of the top-level URLs associated with each website in our data set. The web crawler processes the URL and creates a topographical map of the website, including all its subpages. For instance, a top-level domain, corresponding to a website’s home page, may be associated with the URL “www.healthcare.org.” A subpage of this center might be a page on the team members and associated with the URL “www.healthcare.org/team.” There may be other subpages for specific topics such as the emergency medicine department, the pediatric department, and so forth. Once the web crawler has created a topographical map of a website, that website can then be analyzed for page errors, amount of page content, metadata (ie, titles, keywords, and descriptions), or other preprogrammed factors [25].

Using these usability tools, we scored each website on the four previously mentioned categories. The composite of key factors in each of those categories contributed to an overall “general usability” score for each website. Lastly, an overall score was calculated by applying a weighted percentage across all factors and used for the final ranking system.

Below, we will describe each of the categories we evaluated and their contributed significance.

#### Accessibility

Accessibility is a category that refers to how well a website engages a broad audience with varied levels of technical ability, literacy, and disability. This category includes the following components: meta description, functionality, readability, and the overall layout of the website. A meta description refers to the “snippet” page summary presented in a search engine result. Functionality refers to a website’s ability to provide content that appeals to a broad range of literacy levels. Functionality also includes features that allow users to access different parts of a website. It is estimated that 43% of American adults have basic or below-basic literacy levels [26]. The usability of assistive technologies such as screen readers and magnifiers for websites is also assessed by the accessibility category [27]. In this study, we utilized tools that apply algorithmic scales to rank website reading difficulty and to determine the grade level required to comprehend a website’s content.

#### Content Quality

The content quality category refers to our assessment of the attributes of the content on a website. This can include the relevancy of the written information to that particular point in time on a specific topic, generated metadata, and the use of the website’s multimedia for imagery. For instance, a website dedicated to supplying information on current closed-loop insulin pumps for patients with diabetes may be evaluated on its ability to provide relevant, fact-driven answers to questions that people are seeking answers for (ie, relative costs, ease of use, etc). The multimedia on a website may also be evaluated for issues such as quality (eg resolution) and the available metadata function to add support to the composed content. Content quality also includes written text and may evaluate grammar and spelling.

#### Marketing

The marketing category refers to our assessment of the discoverability of a website, with a particular emphasis on its search engine results pages (SERP), which refers to the websites presented to users when they search for something online using a search engine such as Google. Higher placement in search results can lead to greater visibility, and SERPs are considered by some to be one of the most important elements of digital marketing. The field of search engine optimization (SEO) deals with optimizing a website to place better in SERPs, and effectively implementing SEO may allow health care websites to uphold a corporate image as industry leaders [28]. However, technical SEO auditing, specifically, was beyond the scope of this study.

#### Technology

The technology category refers to our assessment of the technical functionality of a website, as opposed to its content. It evaluates the quality of a website’s technology and technological design and performance, including its front-end design and user experience as well as back-end coding infrastructure and server management. The front end is what the users of the site view when browsing a website. It also involves the analysis of HTML elements to ensure that the user has an easily navigated layout and that the site can be scalable across devices (ie, computers, mobile phones, and tablets). The back end involves the programming code upon which the website runs. This code and other website components, such as its databases, are stored on servers, which functionally allow people to view websites from their own devices. The servers also affect the speed of the site (eg, how quickly it loads for users), which can play a crucial role in gaining and maintaining users and followers. For instance, a previous study conducted by Google [29] showed that a website that takes longer than 3 seconds to load on a mobile device will lose approximately 53% of its users; problematically, that same study revealed that the average mobile website speed is upwards of 18 seconds.

#### General Usability

This was a composite of all the metrics from the prior four categories. This category aims to answer the question, “How good is my website?” This metric may serve as a starting point for health care organizations to perform an initial audit of their website to look for areas of improvement.

#### Overall Usability

An overall usability rank order calculation was included to create a comprehensive evaluation of all major and minor factors across all of the five aforementioned categories. From there, we assigned a percentage weight to create an all-inclusive usability ranking system.

## Results

Scores were assigned to all (N=73) academic medical centers found on the AAHC members list [23].

The category with the highest average score was technology, with 0.82 (SD 0.068, SE 0.008). Accessibility was the second highest scoring subcategory, with an average score of 0.77 (SD 0.059, SE 0.007). The third highest scoring subcategory was marketing with an average score of 0.43 (SD 0.066, SE 0.008). The lowest performing category was content quality, with an average of 0.22 (SD 0.070, SE 0.008). The summary statistics across all five categories are presented in Table 1, and a description of the usability tools used in each of the categories can be found in Multimedia Appendix 1.

The overall rankings for the 73 assessed domains for all categories are reported in Multimedia Appendix 2.

**Table 1:** Academic medical center websites: summary statistics from usability analysis.

Category	Mean (SE)	SD	Minimum	Maximum
Accessibility	0.77 (0.007)	0.059	0.58	0.86
Content quality	0.22 (0.008)	0.069	0.09	0.50
Marketing	0.43 (0.008)	0.066	0.26	0.63
Technology	0.82 (0.008)	0.068	0.66	0.97
General usability	0.62 (0.005)	0.047	0.48	0.71

The top leaders across all usability ranking categories are as follows: (1) accessibility—Duke University; (2) content quality—University of Pittsburgh; (3) marketing—University of Southern California; (4) technology—Rosalind Franklin University of Medicine and Science; and (5) general usability—University of Southern California. The top-performing website in terms of overall usability was that of the University of Southern California.

## Discussion

### Comparison With Prior Work

This study assessed academic medical center websites utilizing the methodology outlined by Calvano et al [24] in a publication that ranked usability of digital health care center websites. Previous studies enabled Calvano et al to assess website usability trends including that of hospitals, digital health centers, and children’s hospitals [3,19,24]. In previous studies, content quality was the highest scoring category. This was postulated to reflect the health care industry’s emphasis on providing adequate information for website users. However, investment in content quality is accompanied by a lower investment in other usability categories.

Another major trend in previous studies was that the technology category was the lowest scoring category [3,20,21,24]. Interestingly, when these study methods were applied to academic medical center websites, we found the opposite. Technology was the highest scoring category, and content quality was the lowest. One possible explanation for this is that the significant amount of time elapsed between the studies enabled website technology to be updated. In addition, compared with community hospitals and digital health centers, academic medical centers are larger institutions with more financial capital to invest in website functionality. Academic medical centers also have more expansive websites, which may cause difficulty in monitoring the quality of the large amount of content produced.

With reference to academic medical centers, we assert that an increased level of importance should be placed on monitoring content quality, thereby ensuring a higher standard of information presented to the general public. As medical advancements continue to become more complex and patients become more comfortable with technology use, a larger number of individuals will turn to the internet for assistance in understanding key medical concepts. Therefore, it is of utmost importance that academic medical centers realize their vital role in providing targeted and relevant content for their website users. This includes providing germane, concise answers to common medical questions people may be searching for to obtain new users and maintain the existing ones.

Another difference between previous studies and our findings involved the accessibility category. In the assessment of children’s hospital websites, previous studies found accessibility to be the lowest scoring category [19]. However, we found accessibility to be the second highest scoring category. One possible explanation for this might be that academic medical centers better understand the importance of creating content that is built at the proper literacy levels and technical complexity to ensure ease of access for a broad audience. Academic medical centers tend to serve as leaders in the medical community and are often sought for guidance by the general public in areas of medical concern, such as the current COVID-19 pandemic. With this in mind, academic medical centers may understand the importance of creating content that is easily comprehended by a range of website users.

Marketing scores were noted to be lower than originally anticipated [19]. Pertaining to health care, marketing is important to ensure users can easily locate an organization’s website within search engine results. It is imperative that academic medical centers employ search engine optimization techniques to enable improved public visibility of their information compared to less authoritative sources.

A specific goal of this research is to promote standardization of website analysis across the health care industry, as it has been neglected previously despite being an important facet of other sectors [12-14]. Recent technological advancements have driven down costs in medicine and increased quality of care [22]. Usability analysis is an important element in this process and enables health care organizations to improve their website presence. With the heightened awareness of technology’s importance in health care due to the COVID-19 pandemic, the web presence of academic medical centers is even more essential to ensuring the proper dissemination of health information.

### Limitations

This study has several limitations. Not all of the social media accounts were directly accessible from the website, making them more difficult to find through Twitter and Facebook’s respective search engines. In most cases, an affiliated Facebook or Twitter page was found. A total of 5 academic medical centers were not associated with a Twitter page, and a total of 2 academic medical centers were not associated with either a Facebook or Twitter page. For the academic medical centers that did not have affiliated Facebook or Twitter pages, this either was because one had not been created for the institution or because a link was not provided on their webpage and was not able to be found using the social media’s search function.

Assessments of a website’s speed can vary depending on the time of the day or the day of data collection. This could be due to changes to the website’s servers, internet connectivity, or computer hardware. To minimize sampling bias, the same computer on the same network was used when measuring parameters such as website speed.

All of this information was collected over the course of several months; therefore, some measures may have changed since initial evaluation.

The AAHC provides their own definition of an academic medical center, which includes three main components: “...an allopathic or osteopathic medical school; one or more other health profession schools or programs…, and one or more owned or affiliated teaching hospitals or health systems” [30]. In accordance with the definition of an academic medical center provided by the AAHC, a clinic webpage would not be considered an academic medical center. However, for consistency of using the full sample of academic medical centers provided on the AACH website, we decided to use clinic webpages for usability analysis if no other institution could be found that better met the definition of an academic medical center. In total, 4 clinic pages were used in the analysis. For example, in the case of Des Moines University’s Osteopathic Medical Center, only the university's clinic page was able to be found via their webpage; therefore, this was the link that was used in the usability analysis.

Our data set was much larger than that of previous studies [21,24] because we analyzed entire institution websites; many of which had between 500,000 and over 1 million URLs. The websites were cut off at 750,000 URLs during web crawling as this was felt to be a sufficient sample size without running into RAM limitations.

### Conclusion

As an increasing number of individuals look to the internet for medical information, responsibility will be placed on academic medical centers to maintain high quality websites, given their status as respected sources of current medical information and research. This study offers an analysis of the overall need for improvement in website usability by academic medical centers. The average general usability score was 0.62, showing the necessity for improving usability measures. Academic medical centers may benefit from taking steps to improve various components of their websites in order to reach their audiences. A suggested step is for these organizations to perform periodic usability audits of their websites to identify areas for improvement. Several of these institutions have significant room for improvement of their overall usability, specifically with content quality and marketing*,* the lowest scoring categories in this analysis. Using content audit tools, institutions can gather data regarding their webpage content and improve upon several factors, including ensuring every webpage has a title of appropriate length, that every webpage has an H1 heading, and that the meta descriptions on the page are concise. Content audits of webpages should be focused on improving the quality of information presented by enhancing aspects such as navigability. Navigability of information can be improved upon by fixing broken web links that are cited on the website, adding alternative text to images, and correctly utilizing keywords on the pages. In terms of marketing*,* we also recommend confirming that Facebook pages and other social media links are highly visible on home screens. In addition, we recommend ensuring that there is enough content on those social media pages. These and other webpage improvements will lead to the enhanced usability of the webpages for patients and academicians alike, thereby furthering the quality of health care information available online.

## Appendix

Multimedia Appendix 1 Defined usability factors with their associated percentage weight, assessment tools, impact, and formulas. Used with permission from Calvano et al [24].

Multimedia Appendix 2 Academic medical center websites and category scores.

## Abbreviations

AAHC: Association of Academic Health Centers
SEO: search engine optimization
SERP: search engine results page
