# Supplementary material for: An Analysis of US Academic Medical Center Websites: Usability Study
Source: J Med Internet Res. 2021 Dec 21;23(12):e27750. doi: 10.2196/27750 (PMC8734930; doi:10.2196/27750)
Supplement: Multimedia Appendix 1 [file jmir_v23i12e27750_app1.docx]

**Multimedia Appendix 1**: Defined usability factors with their associated percentage weight, assessment tools, impact, and formulas. Used with permission from Calvano et al. [24].

| Assessment Factors: | Definition: | Accessibility: | Content Quality: | Marketing: | Technology: | General Usability: | Overall Usability: | Assessment Tools^a^: | Applied Formula^b^: |
| --- | --- | --- | --- | --- | --- | --- | --- | --- | --- |
| Alternative text | Missing image alternative text. This is used to offer a description of an image file contained on a webpage. | 15% |  | 4% | 4% | 5% | 6% | Screaming Frog SEO Spider (Full) | (Total - X) / Total |
| Amount of content | Assesses the amount of words present on an individual webpage. | 8% | 33% | 9% |  | 5% | 9% | Screaming Frog SEO Spider (Full) | Pages >600 / Total |
| Analytics | Utilizes a Google analytics service for tracking website traffic. |  |  | 4% | 4% | 3% | 3% | Browser Developer Tools (Free) | Yes/No |
| Broken Backlinks | Broken inbound backlinks. Backlinks are links from outside websites that link to the website of interest. | 8% |  | 1% | 8% | 5% | 4% | AHREFS (Full) | (Relative High - X) / Relative High |
| Cascading Style Sheets (CSS) | Use of cascading style sheets (CSS) for meeting page design and styling standards. | 15% |  |  | 8% | 4% | 5% | Pingdom Tools (Free) | Yes/No |
| Domain age | The age of registered domain name. |  |  | 4% |  |  | 1% | GoDaddy WHOIS (Free) | Absolute value |
| Error page | Assesses for the number of error pages (4xx) within a given website. |  |  | 4% | 8% | 3% | 3% | Screaming Frog SEO Spider (Full) | (Total - X) / Total |
| Facebook | Number of Facebook likes for a company's social media page. |  |  | 8% |  | 4% | 3% | Facebook (Free) | X / Relative High |
| Headings | Number of missing H1 headers on a website. | 8% |  | 4% | 8% | 5% | 5% | Screaming Frog SEO Spider (Full) | (Total - X) / Total |
| In-line CSS | Assesses for the use of embedded CSS throughout the site for additional page formatting. | 8% |  | 1% | 6% | 5% | 4% | Pingdom Tools (Free) | Yes/No |
| Incoming Backlinks | Number of currently functioning backlinks. |  |  | 9% |  | 5% | 4% | AHREFS (Full) | X / Relative High |
| Meta Data | Number of webpages missing meta descriptions. | 4% | 13% | 7% | 4% | 5% | 6% | Screaming Frog SEO Spider (Full) | (Total - X) / Total |
| Missing Files | Number of missing structured data files. |  |  |  | 8% | 3% | 2% | Screaming Frog SEO Spider (Full) | (Relative High - X) / Relative High |
| Open Graph | Use of Facebook's Open Graph protocol |  |  | 4% | 6% | 4% | 3% | OpenGraphCheck (Free) | Yes/No |
| Popularity | Alexa Popularity Ranking |  |  | 9% |  | 6% | 4% | Alexa Rankings (Free) | (Relative High - X) / Relative High |
| Printability | Whether the website utilizes printer-friendly CSS layouts. |  |  |  | 8% | 5% | 3% | Pingdom Tools (Free) | Yes/No |
| Readability | Assesses the Flesch Kincaid (FK) Reading Ease and Gunning Fog Index (GFI) for reading difficulty and estimated grade level required for understanding. | 8% | 33% | 2% |  | 5% | 7% | Readable (Free) | FK 1/2: Absolute Value  GFI 1/2:(Relative High - X) / Relative High |
| Redirections | Number of redirections (3xx) within a website. | 6% |  |  | 6% | 1% | 2% | Screaming Frog SEO Spider (Full) | (Total - X) / Total |
| Social Interest | Total number of all social media interest (Twitter and Facebook factors combined). |  |  | 11% |  | 5% | 5% | Facebook and Twitter (Free) | Facebook Likes + Twitter Follows |
| Speed | The average website speed across multiple assessment tools. | 6% |  | 3% | 15% | 10% | 7% | Pingdom Tools and Google Pagespeed Insights (Free) | (Relative High - X) / Relative High |
| Spelling | Percentage of spelling errors within a given website. |  | 20% |  |  |  | 2% | Readable (Full) | 100 - X / 100 |
| Twitter | Number of Twitter followers for a company’s social media page. |  |  | 7% |  | 3% | 3% | Twitter (Free) | X / Relative High |
| URL format | Use of URL formats that submit to current SSH protocol. | 7% |  | 7% | 4% | 4% | 5% | MOZ URL Structure (Free) | Yes/No |
| W3C compliance | Number of W3C compliance errors within a website. | 8% |  |  | 8% | 5% | 4% | W3C Markup Validation Service (Free) | (Relative High - X) / Relative High |

^a^Full means that it was the paid, commercially available, version of the product. Free means that it was a free, or open-source.

^b^ "X" indicates the obtained value. Relative indicates that the number is relative to the recorded values across the factor.

**Note**: Percentages do not add to 100 for rounding purposes.
