# Supplementary material for: An Analysis of US Academic Medical Center Websites: Usability Study
Source: J Med Internet Res. 2021 Dec 21;23(12):e27750. doi: 10.2196/27750 (PMC8734930; doi:10.2196/27750)
Supplement: Multimedia Appendix 2 [file jmir_v23i12e27750_app2.docx]

**Multimedia Appendix 2**: Academic medical center websites and category scores

| **Academic Medical Center Website and Link** | **Accessibility** | | **Content Quality** | | **Marketing** | | **Technology** | | **General Usability** | | **Overall Usability** | |
| --- | --- | --- | --- | --- | --- | --- | --- | --- | --- | --- | --- | --- |
|  | **Score** | **Rank** | **Score** | **Rank** | **Score** | **Rank** | **Score** | **Rank** | **Score** | **Rank** | **Score** | **Rank** |
| [Augusta University](https://www.augustahealth.org/) | 0.76 | 47 | 0.22 | 40 | 0.42 | 49 | 0.82 | 39 | 0.60 | 52 | 0.57 | 45 |
| [Baylor College of Medicine](https://www.chistlukeshealth.org/) | 0.76 | 46 | 0.28 | 15 | 0.39 | 56 | 0.80 | 43 | 0.60 | 50 | 0.56 | 50 |
| [Brown University](https://www.rhodeislandhospital.org/) | 0.74 | 53 | 0.15 | 63 | 0.42 | 48 | 0.85 | 28 | 0.62 | 34 | 0.58 | 39 |
| [Des Moines University-Osteopathic Medical Center](https://www.dmu.edu/clinic/) | 0.83 | 17 | 0.26 | 28 | 0.45 | 27 | 0.90 | 13 | 0.65 | 17 | 0.61 | 19 |
| [Duke University](https://www.dukehealth.org/) | 0.86 | 1 | 0.33 | 3 | 0.49 | 7 | 0.88 | 17 | 0.68 | 7 | 0.65 | 5 |
| [East Carolina University](https://www.vidanthealth.com/) | 0.82 | 19 | 0.26 | 24 | 0.46 | 25 | 0.91 | 7 | 0.66 | 13 | 0.62 | 12 |
| [East Tennessee State University](https://www.balladhealth.org/) | 0.85 | 7 | 0.29 | 12 | 0.45 | 26 | 0.76 | 55 | 0.64 | 27 | 0.62 | 15 |
| [Eastern Virginia Medical School](https://www.sentara.com/) | 0.81 | 27 | 0.24 | 34 | 0.48 | 11 | 0.80 | 42 | 0.64 | 26 | 0.61 | 18 |
| [Emory University](https://www.emoryhealthcare.org/) | 0.77 | 39 | 0.18 | 51 | 0.44 | 35 | 0.74 | 65 | 0.62 | 40 | 0.57 | 44 |
| [Florida International University](http://health.fiu.edu/) | 0.73 | 58 | 0.26 | 26 | 0.43 | 39 | 0.78 | 50 | 0.62 | 37 | 0.58 | 40 |
| [Georgetown University Medical Center](https://www.medstargeorgetown.org/) | 0.74 | 56 | 0.26 | 27 | 0.36 | 63 | 0.75 | 61 | 0.54 | 71 | 0.53 | 66 |
| [Health Sciences Center at Prisma Health](https://www.ghs.org/) | 0.67 | 69 | 0.11 | 70 | 0.40 | 53 | 0.84 | 30 | 0.59 | 58 | 0.53 | 63 |
| [Howard University](http://huhealthcare.com/) | 0.68 | 67 | 0.18 | 52 | 0.41 | 50 | 0.86 | 25 | 0.62 | 38 | 0.56 | 49 |
| [Loma Linda University Health](https://lluh.org/) | 0.77 | 37 | 0.18 | 53 | 0.48 | 10 | 0.86 | 22 | 0.67 | 11 | 0.60 | 26 |
| [Louisiana State University Health Sciences Center - Shreveport](https://www.ochsnerlsuhs.org/) | 0.79 | 34 | 0.11 | 72 | 0.35 | 67 | 0.82 | 38 | 0.61 | 42 | 0.55 | 54 |
| [Marshall University](https://www.marshallhealth.org/) | 0.66 | 72 | 0.11 | 71 | 0.27 | 72 | 0.74 | 66 | 0.53 | 72 | 0.47 | 72 |
| [Medical College of Wisconsin](https://www.froedtert.com/) | 0.83 | 10 | 0.25 | 29 | 0.47 | 13 | 0.90 | 12 | 0.67 | 10 | 0.63 | 9 |
| [Medical University of South Carolina](https://muschealth.org/) | 0.67 | 71 | 0.27 | 20 | 0.47 | 19 | 0.77 | 52 | 0.57 | 61 | 0.55 | 56 |
| [Meharry Medical College](https://nashvillegeneral.org/) | 0.77 | 42 | 0.17 | 57 | 0.37 | 60 | 0.90 | 10 | 0.61 | 49 | 0.56 | 52 |
| [Mercer University Health Sciences Center](https://memorialhealth.com/) | 0.73 | 59 | 0.21 | 42 | 0.37 | 61 | 0.76 | 53 | 0.54 | 70 | 0.52 | 67 |
| [Midwestern University](https://www.mwuclinics.com/illinois) | 0.82 | 21 | 0.16 | 61 | 0.29 | 71 | 0.90 | 11 | 0.55 | 67 | 0.54 | 61 |
| [Morehouse School of Medicine](http://morehousehealthcare.com/) | 0.85 | 4 | 0.26 | 23 | 0.42 | 47 | 0.85 | 29 | 0.65 | 20 | 0.62 | 14 |
| [New York Institute of Technology](https://comresearchapp.nyit.edu/clinic/) | 0.67 | 70 | 0.09 | 73 | 0.33 | 69 | 0.74 | 67 | 0.55 | 69 | 0.49 | 71 |
| [Oklahoma State University Center for Health Sciences](https://www.osumc.com/home) | 0.68 | 68 | 0.26 | 25 | 0.39 | 54 | 0.86 | 24 | 0.57 | 63 | 0.54 | 62 |
| [Oregon Health & Science University](https://www.ohsu.edu/health) | 0.83 | 15 | 0.20 | 46 | 0.42 | 42 | 0.79 | 47 | 0.63 | 31 | 0.59 | 32 |
| [Rosalind Franklin University of Medicine and Science](https://www.rfuclinics.com/) | 0.86 | 3 | 0.28 | 18 | 0.35 | 64 | 0.97 | 1 | 0.63 | 33 | 0.59 | 30 |
| [Rutgers Biomedical and Health Sciences](http://www.uhnj.org/) | 0.77 | 40 | 0.21 | 43 | 0.42 | 43 | 0.83 | 36 | 0.64 | 24 | 0.59 | 31 |
| [Stanford University](https://stanfordhealthcare.org/) | 0.58 | 73 | 0.27 | 22 | 0.49 | 8 | 0.74 | 64 | 0.60 | 53 | 0.54 | 60 |
| [State University of New York Downstate Medical Center](https://www.downstate.edu/) | 0.81 | 23 | 0.28 | 17 | 0.41 | 51 | 0.75 | 63 | 0.60 | 51 | 0.57 | 42 |
| [State University of New York Upstate Medical University](https://www.upstate.edu/) | 0.71 | 62 | 0.11 | 69 | 0.37 | 59 | 0.75 | 60 | 0.58 | 60 | 0.52 | 68 |
| [Stony Brook University](https://www.stonybrookmedicine.edu/sbuh) | 0.80 | 31 | 0.24 | 33 | 0.43 | 37 | 0.89 | 15 | 0.65 | 19 | 0.60 | 25 |
| [Temple University](https://www.templehealth.org/) | 0.82 | 22 | 0.18 | 55 | 0.46 | 21 | 0.92 | 5 | 0.67 | 8 | 0.62 | 16 |
| [The Ohio State University](https://wexnermedical.osu.edu/) | 0.81 | 26 | 0.28 | 13 | 0.55 | 4 | 0.91 | 9 | 0.69 | 5 | 0.65 | 4 |
| [The Pennsylvania State University](https://hmc.pennstatehealth.org/) | 0.82 | 18 | 0.28 | 16 | 0.46 | 20 | 0.80 | 44 | 0.65 | 18 | 0.61 | 17 |
| [The University of Arizona Health Sciences Center – Tucson & Phoenix Biomedical Campuses](https://www.bannerhealth.com/) | 0.75 | 50 | 0.29 | 6 | 0.47 | 14 | 0.81 | 41 | 0.63 | 30 | 0.59 | 28 |
| [The University of Iowa](https://uihc.org/) | 0.83 | 14 | 0.21 | 44 | 0.43 | 41 | 0.88 | 16 | 0.64 | 28 | 0.59 | 29 |
| [The University of New Mexico Health Sciences Center](https://hsc.unm.edu/) | 0.77 | 43 | 0.17 | 60 | 0.38 | 57 | 0.76 | 54 | 0.60 | 54 | 0.55 | 58 |
| [The University of North Carolina at Chapel Hill](https://www.unchealthcare.org/) | 0.80 | 29 | 0.20 | 45 | 0.48 | 12 | 0.88 | 18 | 0.65 | 21 | 0.61 | 22 |
| [The University of Tennessee Health Science Center](https://www.lebonheur.org/) | 0.83 | 16 | 0.29 | 10 | 0.54 | 5 | 0.86 | 21 | 0.71 | 2 | 0.67 | 3 |
| [The University of Texas Health Science Center at San Antonio](https://southtexasmed.com/) | 0.81 | 24 | 0.11 | 68 | 0.33 | 68 | 0.93 | 2 | 0.61 | 47 | 0.55 | 53 |
| [Tulane University Health Sciences Center](https://tulanehealthcare.com/) | 0.76 | 44 | 0.29 | 11 | 0.38 | 58 | 0.79 | 46 | 0.57 | 64 | 0.55 | 55 |
| [Uniformed Services University of the Health Sciences](https://tricare.mil/mtf/Walterreed) | 0.86 | 2 | 0.29 | 9 | 0.46 | 24 | 0.87 | 20 | 0.65 | 16 | 0.62 | 11 |
| [University of Arkansas for Medical Sciences](https://uamshealth.com/) | 0.83 | 11 | 0.19 | 49 | 0.44 | 33 | 0.93 | 3 | 0.66 | 12 | 0.61 | 21 |
| [University of California, Davis](https://health.ucdavis.edu/) | 0.70 | 64 | 0.22 | 41 | 0.39 | 55 | 0.72 | 70 | 0.57 | 62 | 0.53 | 65 |
| [University of California, Irvine](http://www.ucihealth.org/) | 0.82 | 20 | 0.18 | 56 | 0.37 | 62 | 0.78 | 48 | 0.61 | 44 | 0.56 | 48 |
| [University of California, San Francisco](https://www.ucsfhealth.org/) | 0.70 | 63 | 0.25 | 31 | 0.58 | 2 | 0.81 | 40 | 0.68 | 6 | 0.63 | 7 |
| [University of Central Florida](https://ucflakenonamedicalcenter.com/) | 0.71 | 60 | 0.15 | 62 | 0.26 | 73 | 0.76 | 57 | 0.48 | 73 | 0.46 | 73 |
| [University of Cincinnati College of Medicine](https://www.uchealth.com/) | 0.76 | 45 | 0.19 | 48 | 0.43 | 36 | 0.83 | 35 | 0.61 | 45 | 0.58 | 38 |
| [University of Colorado](https://www.uchealth.org/) | 0.77 | 38 | 0.12 | 67 | 0.44 | 31 | 0.84 | 32 | 0.62 | 41 | 0.57 | 43 |
| [University of Illinois Chicago](https://uihealth.uic.edu/) | 0.81 | 28 | 0.20 | 47 | 0.42 | 45 | 0.80 | 45 | 0.62 | 35 | 0.58 | 36 |
| [University of Kansas Medical Center](https://www.kansashealthsystem.com/) | 0.85 | 5 | 0.28 | 19 | 0.45 | 28 | 0.77 | 51 | 0.64 | 29 | 0.61 | 20 |
| [University of Louisville](https://uoflhospital.org/) | 0.73 | 57 | 0.13 | 65 | 0.31 | 70 | 0.73 | 68 | 0.56 | 65 | 0.51 | 70 |
| [University of Miami](https://umiamihealth.org/) | 0.79 | 32 | 0.24 | 32 | 0.44 | 30 | 0.84 | 33 | 0.66 | 15 | 0.61 | 24 |
| [University of Minnesota](https://www.mhealth.org/) | 0.69 | 66 | 0.23 | 37 | 0.41 | 52 | 0.66 | 73 | 0.55 | 66 | 0.53 | 64 |
| [University of Missouri-Columbia](https://www.muhealth.org/) | 0.84 | 8 | 0.29 | 8 | 0.47 | 15 | 0.91 | 6 | 0.67 | 9 | 0.63 | 8 |
| [University of Nebraska Medical Center](https://www.unmc.edu/) | 0.74 | 55 | 0.34 | 2 | 0.47 | 17 | 0.67 | 72 | 0.59 | 57 | 0.59 | 33 |
| [University of North Texas Health Science Center at Fort Worth](https://www.unthsc.edu/) | 0.75 | 51 | 0.12 | 66 | 0.35 | 65 | 0.75 | 59 | 0.55 | 68 | 0.51 | 69 |
| [University of Oklahoma Health Sciences Center](https://www.oumedicine.com/) | 0.74 | 54 | 0.17 | 58 | 0.44 | 32 | 0.72 | 71 | 0.61 | 48 | 0.56 | 51 |
| [University of Pennsylvania](https://www.pennmedicine.org/) | 0.75 | 48 | 0.29 | 7 | 0.46 | 23 | 0.76 | 56 | 0.61 | 43 | 0.59 | 35 |
| [University of Pittsburgh](https://www.upmc.com/) | 0.85 | 6 | 0.50 | 1 | 0.57 | 3 | 0.86 | 23 | 0.71 | 3 | 0.68 | 2 |
| [University of Rochester](https://www.urmc.rochester.edu/) | 0.75 | 52 | 0.17 | 59 | 0.42 | 46 | 0.72 | 69 | 0.59 | 59 | 0.55 | 59 |
| [University of Southern California](https://www.keckmedicine.org/) | 0.81 | 25 | 0.25 | 30 | 0.63 | 1 | 0.89 | 14 | 0.73 | 1 | 0.69 | 1 |
| [University of Utah Health Sciences Center](https://healthcare.utah.edu/) | 0.71 | 61 | 0.23 | 36 | 0.46 | 22 | 0.85 | 27 | 0.63 | 32 | 0.58 | 37 |
| [University of Virginia](https://uvahealth.com/) | 0.83 | 13 | 0.24 | 35 | 0.42 | 44 | 0.87 | 19 | 0.64 | 23 | 0.60 | 27 |
| [University of Washington](https://www.uwmedicine.org/) | 0.79 | 35 | 0.18 | 54 | 0.44 | 34 | 0.83 | 34 | 0.62 | 36 | 0.58 | 41 |
| [University of Wisconsin - Madison](https://www.uwhealth.org/) | 0.84 | 9 | 0.31 | 4 | 0.48 | 9 | 0.92 | 4 | 0.69 | 4 | 0.65 | 6 |
| [Vanderbilt University](https://vanderbilthealth.com/) | 0.80 | 30 | 0.19 | 50 | 0.53 | 6 | 0.83 | 37 | 0.66 | 14 | 0.62 | 13 |
| [Virginia Commonwealth University](https://www.vcuhealth.org/) | 0.79 | 33 | 0.30 | 5 | 0.43 | 38 | 0.75 | 58 | 0.61 | 46 | 0.59 | 34 |
| [Wake Forest Baptist Medical Center](https://www.wakehealth.edu/) | 0.77 | 41 | 0.22 | 39 | 0.43 | 40 | 0.75 | 62 | 0.59 | 56 | 0.56 | 47 |
| [Washington University](https://www.barnesjewish.org/) | 0.78 | 36 | 0.28 | 14 | 0.47 | 16 | 0.84 | 31 | 0.64 | 22 | 0.61 | 23 |
| [West Virginia University](https://wvumedicine.org/) | 0.83 | 12 | 0.27 | 21 | 0.47 | 18 | 0.85 | 26 | 0.64 | 25 | 0.62 | 10 |
| [Western University of Health Sciences](https://www.westernuhealth.com/) | 0.75 | 49 | 0.13 | 64 | 0.35 | 66 | 0.91 | 8 | 0.60 | 55 | 0.55 | 57 |
| [Yale University](https://www.ynhh.org/) | 0.69 | 65 | 0.23 | 38 | 0.44 | 29 | 0.78 | 49 | 0.62 | 39 | 0.57 | 46 |
